# Animal-derived natural products for hepatocellular carcinoma therapy: current evidence and future perspectives

**DOI:** 10.3389/fphar.2024.1399882

**Published:** 2024-05-09

**Authors:** Yichao Liao, Feng Wei, Zhelin He, Jingxue He, Yanlin Ai, Cui Guo, Li Zhou, Dan Luo, Chengen Li, Yueqiang Wen, Jinhao Zeng, Xiao Ma

**Affiliations:** ^1^ Department of Gastroenterology, Hospital of Chengdu University of Traditional Chinese Medicine, Chengdu, China; ^2^ School of Clinical Medicine, Chengdu University of Traditional Chinese Medicine, Chengdu, China; ^3^ TCM Regulating Metabolic Diseases Key Laboratory of Sichuan Province, Hospital of Chengdu University of Traditional Chinese Medicine, Chengdu, China; ^4^ Endoscopy Center, Guang’an Hospital of Traditional Chinese Medicine, Guang’an, China; ^5^ School of Basic Medicine, Chengdu University of Traditional Chinese Medicine, Chengdu, China; ^6^ State Key Laboratory of Southwestern Chinese Medicine Resources, School of Pharmacy, Chengdu University of Traditional Chinese Medicine, Chengdu, China; ^7^ School of Pharmacy, Chengdu University of Traditional Chinese Medicine, Chengdu, China

**Keywords:** anti-hepatocellular carcinoma, natural products, animal-derived natural products, animal medicines, mechanisms

## Abstract

Hepatocellular carcinoma (HCC) has a high morbidity and mortality rate, and the survival rate of HCC patients remains low. Animal medicines have been used as potential therapeutic tools throughout the long history due to their different structures of biologically active substances with high affinity to the human body. Here, we focus on the effects and the mechanism of action of animal-derived natural products against HCC, which were searched in databases encompassing Web of Science, PubMed, Embase, Science Direct, Springer Link, and EBSCO. A total of 24 natural products from 12 animals were summarized. Our study found that these natural products have potent anti-hepatocellular carcinoma effects. The mechanism of action involving apoptosis induction, autophagy induction, anti-proliferation, anti-migration, and anti-drug resistance via phosphoinositide 3-kinase (PI3K)/protein kinase B (Akt)/mammalian target of rapamycin (mTOR), Ras/extracellular signal regulated kinases (ERK)/mitogen-activated protein kinase (MAPK), Wnt/β-catenin, and Janus kinase (JAK)/signal transducer and activator of transcription (STAT) pathways. Huachansu injection and sodium cantharidate have been used in clinical applications with good efficacy. We review the potential of animal-derived natural products and their derivatives in the treatment of HCC to date and summarize their application prospect and toxic side effects, hoping to provide a reference for drug development for HCC.

## 1 Introduction

In the world, liver cancer ranks second in terms of mortality and is the sixth most frequent cancer. GLOBOCAN data indicates that in 2019, there were over 900,000 new cases of liver cancer worldwide, resulting in 830,000 deaths, posing a significant health threat (Cao et al., 2021; Sung et al., 2021; Peng et al., 2023). The most common cause of liver cancer is infection with the hepatitis virus; other important risk factors include obesity, diabetes, alcohol usage, and aflatoxin (Sayiner et al., 2019). Ninety percent of liver cancer cases are attributed to hepatocellular carcinoma (HCC), which is the predominant kind of liver cancer (Qin et al., 2023). Currently, surgical resection, chemotherapy, liver transplantation, percutaneous ablation, transarterial chemoembolization, and transarterial radioembolization are the main treatments for HCC (Chen et al., 2023). The majority of HCC patients, however, do not have the opportunity for surgical therapy at the time of diagnosis because of the disease’s hidden start, which leaves them with a median survival duration of roughly 6–8 months (Asrani et al., 2019). The most popular treatment for HCC is chemotherapy, yet its efficacy is restricted by its toxicity and drug resistance. New therapeutic approaches are desperately needed right now to enhance the treatment status of HCC, and finding safer medications with more potent targeting for HCC is seen to have a great deal of potential.

Over 80% of people worldwide predominantly use natural products for treating illnesses, according to the WTO (Chen T. et al., 2024; Deng et al., 2024; Luo et al., 2024). Apart from plants and their derivatives, animals and their derivatives also have excellent therapeutic effects when used as natural medicines (González and Vallejo, 2021). Over 1,500 different kinds of animal medicines are employed in traditional Chinese medicine; at least 584 different animal species are used for medical purposes in Latin America; and 29 monographs titled “Russian Pharmacopoeia” describe medications produced from animals (Prokopov et al., 2019). In Indian Unani medicine, approximately 200 kinds of medicines are derived from animals (Mahawar and Jaroli, 2008). C-nucleosides discovered from Caribbean marine sponges are important materials for synthesizing cytarabine, which has been one of the most effective chemotherapy drugs for treating acute myeloid leukemia since the 1960s (Saini et al., 2023).

Animal medicines typically contain a wide range of biologically active chemicals with a high affinity for the human body as compared to other medications. Recently, the particular phrase “animal therapy” has been proposed, meaning that human diseases can be treated with remedies obtained from or derived from animals (Valiakos et al., 2021). This method is becoming a crucial component of alternative therapies. Animal medicines are predicted to rank among the most promising medications for the treatment of cancer as a result of increasing research into them and the elucidation of their applications and mechanisms. In this review, we recall animal-derived natural products with the potential for treating HCC and discuss their potential targets and mechanisms of action provide guidance and preclinical evidence for further studies of animal-derived natural products for the treatment of HCC. It is expected to provide new approaches of drug development for further translation into the clinical setting.

## 2 Methods

**FIGURE 1:**
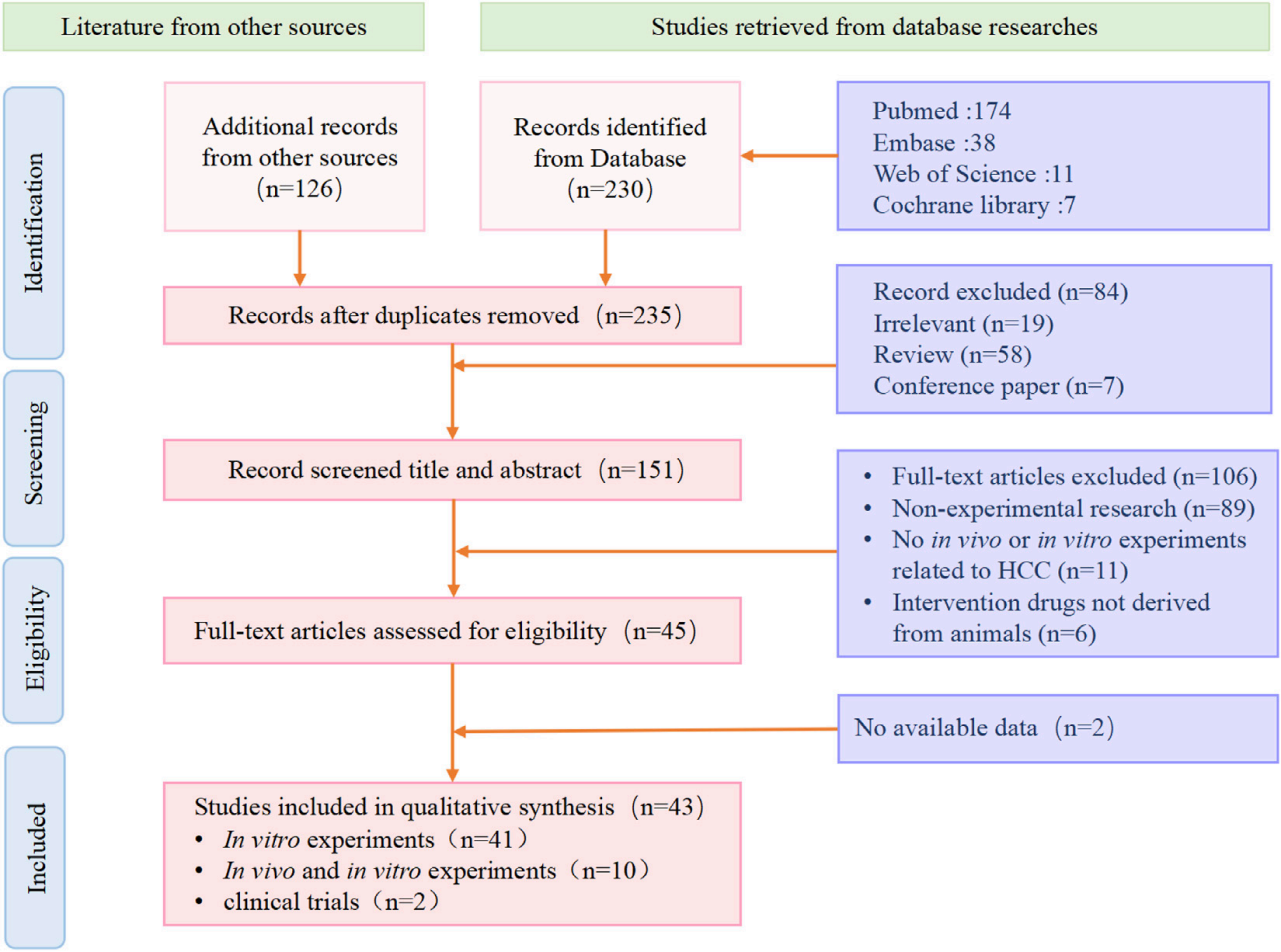
We show the process of inclusion and exclusion of literature in the systematic review. Screening of literature after retrieval, including a total of 43 eligible articles according to the criteria.

We conducted a comprehensive search of literature published from the inception of the databases to 1 September 2023, in Web of Science, Pubmed, Embase, Springer Link, Science Direct, and EBSCO, and organized and analyzed the literature. We used a strategy of subject words + free words to search for literature. The search strategy mainly included two parts: (1) “liver cancer”, (2) “animal-derived products”. The inclusion criteria for the literature were: (1) experimental research; (2) *in vivo* or *in vitro* models of hepatocellular carcinoma; (3) at least one intervention group used animal extracts. The exclusion criteria for the literature were: (1) non-experimental research; (2) no *in vivo* or *in vitro* experiments related to HCC; (3) intervention drugs not derived from animals; (4) experiments that did not meet ethical requirements or were not peer-reviewed (Figure 1).

## 3 Result

A total of 356 articles were retrieved from multiple databases. After excluding non-experimental articles and duplicate articles, 235 studies were initially included. After reading the full text, a further selection resulted in 43 studies. We organized these results according to the species category of the drug source. These studies confirmed the potential of natural animal medicines against liver cancer. They inhibited the growth, migration, and other malignant behaviors of liver cancer through multiple different molecular mechanisms, and promoted the apoptosis of liver cancer cells (Table 1).

**TABLE 1:** Animal-derived natural products for hepatocellular carcinoma therapy.

Compound	Source	Experimental model		Efficacy	Mechanism	References
		*In vitro*	*In vivo*			
Cinobufotalin	*Bufo gargarizans* Cantor or *Bufo melanostictus* Schneider	HepG2; SMMC-7721; SUN-368; KYN-2; Huh-7	Balb/c mice injected with H22 cells; SMMC-7721 xenograft mice	Induction of apoptosis; Anti-migration and proliferation	β-catenin↓; E-cadherin↑ N-cadherin↓; PP2A↑; PP1A↑ ACC↓; ACLY↓; FASN↓; SCD1↓; EMT (−); mTORC (−)	(Ogretmen and Hannun, 2001; Cheng et al., 2015; Giannelli et al., 2016; Meng et al., 2021; Li et al., 2022)
Cinobufagin		Huh-7 HepG2 SK-HEP-1	Huh-7 and Huh-7/AURKA xenograft mice	Induction of apoptosis; Induction of cell cycle arrest	BCL-2/BAX↓; CDK1↓; Fas↑; cyclinB1↓; PCNA↓; CDK1↓; Caspase-9↑; Caspase-3↑; Caspase-8↑; Caspase-10↑; p53↑; p73↓; AURKA-mTOR-eIF4E (−)	(Qi et al., 2011; Zhao et al., 2019a; Zhang et al., 2020a; Jin et al., 2020)
Bufotalin		Hep 3B; R-HepG2	R-HepG2 xenograft mice	Induction of apoptosis; Induction of cell cycle arrest; Anti-drug resistance	Caspase-8↑; Caspase-9↑; Caspase-3↑; AuroraA↓; CDC25↓; CDK1↓; cyclinB↓; p53↑; p21↑ BAX↑; BCL-2↓; Akt (+)	(Su et al., 2009; Zhang et al., 2012)
Bufalin		HCCLM3; HepG2; SK-Hep-1; Huh7; Hep3B; HA22T; Bel-7402	HCCLM3-R xenograft mice	Induction of apoptosis; Anti-migration and proliferation; Induction of cell cycle arrest; Induction of cellular autophagy	β-catenin↓; E-cadherin↑; MMP-2↓; MMP-9↓; beclin-1↑; CHK1↑ p62↓; AMPK↑; Akt↓; cyclin A↓; cyclin B↓; AFP↑; CDK1↓; WEE1↑; Lc3-Ⅱ↑; CDC↑; CDC25C↑; TNF-α↑; ATG8 family↑; ALB↑; Akt/GSK3β/β-catenin/E-cadherin (+); mTOR (−); JNK/MAPK8 (+); EMT (−); Akt/mTOR (−)	(Tsai et al., 2012; Hsu et al., 2013; Miao et al., 2013; Qiu et al., 2013; Zhang et al., 2014; Gai et al., 2016)
Gepsin	Gekko swinhonis Guenther	Bel-7402		Induction of cell cycle arrest	AFP↓; ALB↑	Wu et al. (2006)
GSPP		SMMC-7721		Anti-migration and proliferation; Induction of cell cycle arrest	Ca2+↓	(Chen et al., 2010; Wu et al., 2011)
GSPP α		SMMC-7721		Anti-migration and proliferation; Induction of cell cycle arrest	IL-8↓; Ca2+↓	(Chen et al., 2010; Wu et al., 2011)
Cantharidin(CTD)	Mylabris phalerata Pallas or Mylabris cichorii Linnaeus	HepG2; Hep3B; SMMC-7721	HepG2 xenograft mice	Induction of apoptosis; Anti-drug resistance	P-gp↓; MDR1↓; JAK2/STAT3 (−); P13K/Akt (−)	(Zheng et al., 2008; Zhu et al., 2020)
Norcantharidin(NCTD)		HepG2; SMCC-7721; MHCC-97H; Hep3B	mice were injected with DEN to initiated HCC; Balb/c mice injected with H22 cells	Induction of apoptosis; Induction of cell cycle arrest	ROS↑; cyclin B↓; CDK1↓; BCL-2↓; BAX↑; miR-214↑; Caspase-3↑; Caspase-10↑; β-catenin↑; MEK (−); BCL-2/Caspase (+); Ras (−); ERK (−); STAT3 (−)	(Chang et al., 2010; Li et al., 2010; Yeh et al., 2012; Lu et al., 2014; Zhang et al., 2017)
Methyl-cantharidimide(MCA)		BEL-7404; HepG2		Induction of apoptosis; Induction of cell cycle arrest	Caspase-3↑; p53-UNC5B (+); NF-κB (−)	(Wei et al., 2012; Li et al., 2020)
Beauvericin	*Bombyx mori* Linnaeus	H4IIE		Induction of apoptosis	E-cadherin↑; N-cadherin↓; JNK (+); ERK (−); NF-κB (−); EMT (−)	Wätjen et al. (2014)
BBPW-2		HepG2		Induction of apoptosis; Induction of cell cycle arrest	\	Jiang et al. (2014)
Echinoside A (EA)	Pearsonothuria graeffei Semper	HepG2		Anti-migration and proliferation	MMP-9/TIMP-1↓; VEGF↓	(Zhao et al., 2011; Wang et al., 2014)
Holothurin A1 (HA1)		HepG2	CAM model	Anti-migration and proliferation	MMP-9↓; TIMP-1↑	Zhao et al. (2010)
24-Dehydroechinoside A (DHEA)		HepG2	CAM model	Anti-migration and proliferation	MMP-9↓; TIMP-1↑	Zhao et al. (2010)
Sipholenol A	Siphonochalina	HepG2		Induction of apoptosis; Induction of cell cycle arrest; Anti-drug resistance	cyclin-B1↓; cyclin-D1↓; Caspase-3↑	Abdel-Lateff et al. (2016)
Crambescidin-816 (C816)		HepG2		Anti-migration and proliferation; Induction of cell cycle arrest	cyclin-A↓; cyclin-D ↓; kinases2↓; kinases6↓	Rubiolo et al. (2014)
Aaptamine		HCC-LM3; HepG2	HCC-LM3 tumor xenografts mice	Induction of cell cycle arrest	SOX9↓; CDK2↓	Li et al. (2015)
Bromovulone III	Alcyoniidae	Hep3B; HepG2; HA22T		Induction of apoptosis	m-calpain↑; Caspase-7/12↑; CHOP/GADD153↑	Chiang et al. (2005)
Methyl spongoate (MESP)		BEL-7402; SMMC-7221; Zip-177; HepG3B; HepG2; BEL-7404		Induction of apoptosis	MMPs↓; Caspase-9/3↑; BAX↑; Caspase-3/9↑ STAT3 (+)	(Jiang et al., 2010; Jiang et al., 2011)
11-epi-sinulariolide acetate (11-epi-SA)		HA22T; Hep3B; Huh7		Induction of apoptosis; Anti-migration and proliferation	MMP-2↓; MMP-9↓; uPA↓; TIMP-1↑; TIMP-2↑; BAX↑; BAD↑; BCL-2↑ ERK (−); p38MAPK (−); FAK/P13K/Akt/mTOR (−)	(Lin et al., 2014; Lin et al., 2016)
Extractive of Periplaneta american Linneaus	Other animal-derived natural products	HepG2		Induction of apoptosis	P13K/Akt (−)	Ma et al. (2022)
Kahalalide F (KF)		—		Induction of cell cycle arrest	TGF-α (−)	Salazar et al. (2013)
β-chitosan nanoparticles (β-CNP)		HepG2		Induction of apoptosis	mitochondrial pathway (+)	Subhapradha and Shanmugam (2017)

All 43 experimental studies included in the full review were peer-reviewed for publication. Of the 43 experimental studies, 41 were *in vitro* experiments and 2 were clinical trials, of which 10 were both *in vitro* and *in vivo* experiments. The *in vitro* trials were randomised and controlled, which prevents factors other than the study intervention from influencing the results, thus improving the accuracy of the results. 2 clinical trials were conducted, which provided us with clinical data.

### 3.1 *Bufo gargarizans* cantor or *Bufo melanostictus* schneider

The *Bufo gargarizans* Cantor or *Bufo melanostictus* Schneider also called toad, belongs to the Bufonidae family, and is mainly distributed in East Asia. ChanSu is the dried skin secretion of the Asiatic toad (*B. gargarizans* Cantor) or black-spectacled toad (*B. melanostictus* Schneider). Modern research shows that it has various pharmacological activities such as cardiotonic, antitumor, analgesic, anti-inflammatory, and immune regulation (Zhan et al., 2020). Bufadienolides are the most important bioactive substances in Chan Su, including bufalin, bufotalin, cinobufagin, cinobufotalin, telocinobufagin, etc (Jia et al., 2022). They can effectively combat various cancers such as liver cancer, osteosarcoma, colorectal cancer, gastric cancer, lung cancer, and breast cancer. Huachansu is an injection made from further processing of Chan Su and is widely used in China for the treatment of tumors. A clinical trial included 15 patients with stage III or IV hepatocellular carcinoma, non-small cell lung cancer, or pancreatic cancer. After two cycles of high-dose Huachansu intravenous administration, no dose-limiting toxicity appeared, and the tumors in 6 patients were stable or slightly reduced (Meng et al., 2009).

#### 3.1.1 Cinobufotalin

Cinobufotalin is a bufadienolide isolated from toad venom and has been proven to have strong antitumor effects (Chen and Kovaríková, 1967). Studies have found that 80 nM of cinobufotalin is toxic to HepG2, SMMC-7721, and SNU-368 cells, and it inhibits their epithelial-mesenchymal transition (EMT), weakening the migration and invasion of HCC and inhibiting tumor growth. Further research shows that cinobufotalin can inhibit the level of β-catenin, increase the expression level of E-cadherin, and decrease the level of N-cadherin, thereby inhibiting the EMT of HCC. In the BALB/c mouse tumor model, it was verified that the inhibition of EMT by cinobufotalin depends on the downregulation of β-catenin, and no obvious toxic reactions were observed (Li et al., 2022). EMT is the process by which epithelial cells lose their epithelial characteristics and gain mesenchymal properties, and it plays an important role in the early stage of liver cancer metastasis (Giannelli et al., 2016).

Another study found that 250 nM of cinobufotalin could promote apoptosis in KYN-2, HepG2, and Huh-7 cells. This might be achieved by inhibiting the activity of sphingosine kinase 1 (SPHK1) and increasing the accumulation of ceramide in cells (Cheng et al., 2015). The excessive accumulation of ceramide can increase the activity of serine/threonine phosphatases (PP) such as PP2A and PP1A, leading to the dephosphorylation and inactivation of protein kinase B (Akt), inhibition of the mammalian target of rapamycin (mTOR) pathway, and thus inducing apoptosis in liver cells (Ogretmen and Hannun, 2001).

Cinobufotalin can also selectively block the interaction of sterol regulatory element-binding protein 1 (SREBP1) with sterol regulatory elements (SRE) in HepG2, LM3, and SMMC7721 cells, reducing the transcriptional activity of SREBP1, thereby inhibiting the expression of fat generation-related genes acetyl-CoA carboxylase (*ACC*)*,* ATP citrate lyase (*ACLY*)*,* fatty acid synthase (*FASN*)*,* Stearoyl-CoA desaturase-1 (*SCD1*), and treating HCC by inhibiting *de novo* fat synthesis (Meng et al., 2021). The lipid metabolism disorder of tumor cells seems to contribute to their growth, and blocking *de novo* lipid biosynthesis to treat tumors has great research prospects (Pope et al., 2019; Guo et al., 2020).

#### 3.1.2 Cinobufagin

Cinobufagin is also a bufadienolide isolated from Chan Su and is a Na^+^/K^+^-ATPase inhibitor with strong anticancer effects. Studies have found that 0.1 μmol/L of cinobufagin can effectively inhibit the activity of HepG2 cells and induce apoptosis through endogenous and exogenous pathways. Cinobufagin can upregulate Bcl2 associated x (BAX) expression and downregulate Bcl2 apoptosis regulator (BCL-2) expression, activate cysteine-dependent aspartate-directed proteases (Caspase) family: Caspase-9, Caspase-3, and poly ADP-ribose polymerase (PARP)-mediated apoptosis. At the same time, cinobufagin can also upregulate Fas expression, activate Caspase-8 and Caspase-10, and induce apoptosis through the mitochondrial pathway (Qi et al., 2011).

Another study found that after 24 h of treatment with 5 μmol/L of cinobufagin, the activity of Huh-7 cells could be inhibited by 50%. Cinobufagin can reduce the BCL-2/BAX ratio in an aurora kinase A (AURKA)-dependent manner, inducing apoptosis in Huh-7 cells. At the same time, cinobufagin can also inhibit the expression of cyclin-dependent kinase (CDK)1, cyclinB1, and proliferating cell nuclear antigen, causing cell cycle arrest in the G2/M phase. Cinobufagin inhibits the vitality of Huh-7 cells by inhibiting AURKA and p53 signal transduction and activating p73 signal transduction, thereby blocking the cell cycle and inducing cell apoptosis (Zhao L. et al., 2019).

Several studies have validated the effects of cinobufagin on HCC. Cinobufagin at 125 nM has a strong growth inhibitory effect on HepG2 and SK-HEP-1 cells. Cinobufagin inhibits the growth of HCC cells by suppressing the expression of epidermal growth factor receptor and reducing the activity of CDK2 (Zhang Q. et al., 2020). Another study found that cinobufagin can inhibit the AURKA-mTOR-eukaryotic translation initiation factor 4E (EIF4E) signaling pathway, and block the formation of the spindle body in Huh-7 cells, thereby exerting an anti-tumor effect (Jin et al., 2020).

#### 3.1.3 Bufotalin

Bufotalin is also a bufadienolide that can be used for heart failure and pain (El-Seedi et al., 2022). One study found that 1.13 μM of bufotalin has an 80% growth inhibition rate on Hep 3B cells. After bufotalin intervention, the expression of Caspase-8 in Hep 3B cells is upregulated, which subsequently activates the Caspase-9 and Caspase-3 pathways, leading to PARP cleavage and inducing cell apoptosis through the mitochondrial pathway (Su et al., 2009).

In addition, 2 nM of bufotalin can inhibit the growth of multi-drug resistant liver cancer cells R-HepG2 and suppress the cell cycle in the G2/M phase *in vitro* and *in vivo*. Further research found that bufotalin can downregulate the expression of Aurora A and cell division cycle (CDC)25, thereby inhibiting CDK1 and cyclinB, inducing cell stagnation in the G2/M phase. At the same time, bufotalin can also increase p53 expression, on one hand, it can block the cell cycle by promoting the expression of cyclin-dependent kinase inhibitor 1 (p21); on the other hand, it can upregulate BAX and downregulate BCL-2, inducing mitochondrial pathway apoptosis signals. The study also found that bufotalin can inhibit the expression and phosphorylation of Akt, promoting p53-mediated cell apoptosis (Zhang et al., 2012).

#### 3.1.4 Bufalin

Bufalin is a soluble digitalis-like component in Chan Su. Research has found that 10 nmol/L of bufalin can significantly inhibit the proliferation, migration, and invasion of HCCLM3 and HepG2 cells. Molecular experiments show that bufalin can inhibit the phosphorylation of Akt and increase the activity of glycogen synthase kinase-3 (GSK3)β, thereby inducing the degradation of β-catenin. This leads to the subsequent inhibition of matrix metalloproteinase (MMPs), such as MMP-2 and MMP-9 expression and an increase in E-cadherin expression (Qiu et al., 2013). *In vivo* experiments show similar results, bufalin can inhibit tumor growth in mice with orthotopic transplantation tumor models of HCC through the Akt/GSK3β/β-catenin/E-cadherin pathway, promoting tumor necrosis (Zhang et al., 2014).

Research has also found that bufalin can inhibit the proliferation of HepG2 liver cancer cells in a dose-dependent manner, with an IC50 value of 143.2 nM. Further research has found that it increases the expression of beclin-1, promotes the transformation of Lc3-I to Lc3-II, and reduces the expression of sequestosome-1 (p62), initiating and forming autophagosomes (Chen Y. et al., 2024), and inducing cell autophagy. At the same time, bufalin can also activate AMP-activated protein kinase activity, thereby inhibiting mTOR to induce autophagy. Autophagy leads to the degradation of the main contents of the cytoplasm by autophagic lysosomes, abnormal protein aggregation, and excess and damaged organelles, which have a dual role in promoting survival and apoptosis (Miao et al., 2013).

In addition, bufalin can reduce Akt activity, interfere with the Akt/mTOR signaling pathway, and simultaneously increase the protein levels of LC3-II, autophagy-related proteins, and beclin-1 in SK-Hep-1 cells, collectively inducing autophagy in SK-Hep-1 cells. Bufalin intervention also reduces the expression of cyclin A, cyclin B, and CDK1 in SK-Hep-1 cells, and increases the expression of checkpoint kinase 1 (CHK1), and wee1-like protein kinase (WEE1), causing cell cycle arrest in the G2/M phase (Tsai et al., 2012). Another study showed similar results, apoptotic protease activating factor-1 can significantly inhibit the proliferation of Huh7, Hep3B, and HA22T cells after 0.4 μM of bufalin acting for 72 h, by up-regulating the expression of cyclin B, CDC, and CDC25C, causing cell cycle arrest in the G2/M phase, and by activating tumor necrosis factor (TNF)-α, c-Jun N-terminal kinase (JNK)/mitogen-activated protein kinase 8 (MAPK8), beclin-1, and gamma-aminobutyric acid receptor-associated protein, etc., promoting cell autophagy (Hsu et al., 2013).

Bufalin can inhibit the proliferation, migration, and invasion of Bel-7402 cells. After treating BEL-7402 cells with 0.085 μg/mL of bufalin for 72 h, the number of migrations and invasions decreased by more than 50%. Further experimental results show that bufalin can increase the expression of β-catenin on the cell membrane, reduce the expression of β-catenin in the cytoplasm and nucleus, and inhibit EMT by increasing the expression of E-cadherin. At the same time, it can also reverse the malignant phenotype of cells by reducing the expression of alpha-fetoprotein (AFP) in BEL-7402 cells and increasing the expression of albumin (ALB) (Gai et al., 2016).

### 3.2 *Gekko swinhonis* guenther

*Gekko swinhonis* Guenther, also known as the Gecko, is an animal of the genus *Gekko*, family Gekkonidae. In China, the dried body of the gecko has been used as an anti-cancer traditional medicine for hundreds of years. Modern research shows that various compounds extracted from gecko, such as gecko polypeptides, have various biological effects, such as anti-inflammatory and anti-tumor, and are very promising anti-cancer drugs (Duan et al., 2018; Babaei et al., 2023).

#### 3.2.1 Gepsin

Gekko sulfated polysaccha-ride (Gepsin) is a sulfated polysaccharide isolated from the gecko. At 10 mg/mL, Gepsin can inhibit Bel-7402 cell proliferation by 50%, but it has no discernible effect on normal liver L-02 cells. Gepsin can suppress the cell cycle in the G2/M phase, but it has no discernible effect on cell apoptosis. In addition, after Gepsin treatment, the secretion of AFP in HCC cells decreases, and the secretion of ALB increases, indicating that Gepsin can inhibit the malignant differentiation of HCC cells (Wu et al., 2006).

#### 3.2.2 GSPP and GSPP *α*

The gekko sulfated polysaccharide-protein complex (GSPP) isolated from the gecko also has certain anticancer effects. GSPP can inhibit the proliferation of SMMC-7721 cells, cause cell cycle arrest in the S phase, and inhibit the migration of SMMC-7721 cells by reducing the intracellular calcium concentration and inducing the aggregation of actin filaments and the redistribution of actin filaments to the cytoplasm (Chen et al., 2010). Subsequently, through papain hydrolysis, a substance with a lower molecular weight and better antitumor activity—gekko sulfated glycopeptide α (GSPPα)—was extracted from the GSPP. GSPPα can inhibit the migration of SMMC-7221 cells in a dose-dependent manner by reducing the secretion of interleukin-8 (IL-8) and the intracellular calcium concentration and regulating the reorganization of the cell skeleton (Wu et al., 2011).

### 3.3 *Mylabris phalerata* Pallas or *Mylabris cichorii* Linnaeus

*Mylabris phalerata* Pallas*,* or *Mylabris cichorii* Linnaeus, also called the Chinese blister beetle, is a common insect in China. Traditional Chinese medicine uses the dried body of this insect to cure a variety of cancers and skin conditions (Wang, 1989; Knapp et al., 1998; Li et al., 2023). The blister beetle has significant toxicity to the liver, kidneys, gastrointestinal tract, and heart, but various active ingredients extracted from it have significant therapeutic effects on liver cancer, skin cancer, breast cancer, bladder cancer, lung cancer, colorectal cancer, and more (Naz et al., 2020). A medication called sodium cantharidate, which is derived from blister beetle extract, has a definite therapeutic effect on liver cancer. A clinical trial that included 104 patients with advanced HCC found that, compared to conventional treatment regimens, treatment with sodium cantharidate had a higher rate of alleviation and stability (Shao et al., 2014).

#### 3.3.1 Cantharidin

Cantharidin (CTD) is an active natural product extracted from the blister beetle which can induce apoptosis in various types of tumor cells (Deng et al., 2013). Research has found that 5 μM of CTD can significantly reduce the proliferation of HepG2 and Hep3B cells. Experiments have confirmed that EPHB4 is the main target of CTD. CTD inhibits ephrin type-B receptor 4 (EPHB4), blocking Janus kinase (JAK)2/signal transducer and activator of transcription (STAT)3 and phosphoinositide 3-kinase (PI3K)/Akt pathways, and thereby promoting apoptosis of HCC cells through endogenous pathways. *In vivo* experiments also confirmed that CTD inhibits the progression of SMMC-7721 xenograft tumors in mice by blocking the EPHB4-related pathway (Zhu et al., 2020).

Another study found that CTD can inhibit the multidrug resistance (MDR) of HepG2/ADM cells by suppressing the levels of p-glycoprotein (P-gp) and MDR1. The overexpression of P-glycoprotein and MDR1 is a major cause of chemotherapy resistance in tumor cells., P-gp-mediated drug efflux inhibition causes MDR cancer cells to become resensitized to chemotherapeutic agent treatment, which may enable chemotherapy to be successfully administered to patients with MDR tumors (Zheng et al., 2008).

#### 3.3.2 Norcantharidin

Norcantharidin (NCTD) is a demethylated analogue of CTD, which can effectively combat tumors. NCTD at 5–40 μg/mL can inhibit the proliferation of HepG2 cells in a dose-dependent manner. Further research has found that NCTD can induce apoptosis in HepG2 cells through the BCL-2/Caspase pathway (Chang et al., 2010). At the same time, NCTD can increase the reactive oxygen species level in HCC cells, promoting mitochondrial damage and cell apoptosis (Li et al., 2010).

In addition, 10 μg/mL of NCTD can significantly inhibit the proliferation of SMCC-7721 and MHCC-97H cells, which is related to upregulation of terminal nucleotidyltransferase 5C (FAM46C) expression by NCTD. FAM46C is a non-canonical poly(A) polymerase that can inhibit the activity of Ras, mitogen-activated protein kinase (MEK), and extracellular signal regulated kinases (ERK), thereby inhibiting the expression of cyclin B, CDK1, BCL-2, promoting the expression of BAX, inducing cell cycle arrest in the G2/M phase, and cell apoptosis (Zhang et al., 2017). NCTD also promotes the expression of miR-214, thereby inhibiting the β-catenin pathway and STAT3 pathway, exerting anti-cancer effects (Lu et al., 2014).

The anti-cancer mechanism of NCTD may be related to the dose. Research has found that 25 μg/mL of NCTD can induce cell cycle arrest in the G2/M phase in Hep G2 cells, and 50 μg/mL of NCTD can induce G0/G1 phase arrest. In addition, NCTD can also bind with the TNF-related apoptosis-inducing ligand (TRAIL)-R2/DR5. TRAIL-R2/DR5 is a type of TRAIL receptor that can activate Caspase-3 and Caspase-10, thereby inducing cell apoptosis (Yeh et al., 2012).

#### 3.3.3 Methyl-cantharidimide

Methyl-cantharidimide (MCA) is a CTD analogue, possessing stronger anti-tumor effects and lower toxicity than CTD. MCA can promote apoptosis in BEL-7404 and HepG2 cells, and inhibit cell growth, with IC50 values of 226.82 and 273.18μM, respectively (Wei et al., 2012). MCA can increase the expression of the netrin receptor UNC5B (UNC5B) in HCC cells, activating apoptosis mediated by p53-UNC5B. In addition, MCA can also inhibit the expression of nuclear factor-κB (NF-κB), increase the level of Caspase-3, thereby exerting anti-HCC effects (Li et al., 2020).

### 3.4 *Bombyx mori* Linnaeus

The *Bombyx mori* Linnaeus commonly known as silkworm and belongs to the family of Bombycidae. In China, the dried body of silkworm larvae infected with beauveria bassiana is known as “Jiangcan”, which can be used to treat diseases such as stroke and inflammation. Modern research has confirmed that this special silkworm has certain anti-tumor effects, and it has certain effects on osteosarcoma, liver cancer, and cervical cancer (Wu et al., 2011; Yue et al., 2009).

#### 3.4.1 Beauvericin

Beauvericin is a common mycotoxin, and studies have found that it can induce apoptosis in H4IIE cells. Further research shows that Beauvericin can reduce the phosphorylation of ERK, induce the phosphorylation of JNK, and inhibit the activity of NF-κB, thereby inducing cell apoptosis (Wätjen et al., 2014).

#### 3.4.2 BBPW-2

BBPW-2 is a water-soluble oligosaccharide purified from B. batryticatus. BBPW-2 at a concentration of 0.5 mg/L can effectively inhibit the growth of HepG2 cells, causing cell cycle arrest in the G0/G1 and G2/M phases, and promoting cell apoptosis (Jiang et al., 2014).

### 3.5 *Pearsonothuria graeffei* Semper

*Pearsonothuria graeffei* Semper belongs to Holothuriidae, which also known as sea cucumber. Holothuriidae has been used as a source of anti-inflammatory and anti-disease food for centuries (Hossain et al., 2020). Sea cucumbers consist of vitamins, minerals, cerebrosides, peptides, and lectins, and also contain unique molecules, such as sulfated polysaccharides, 12-methyltetradecanoic acid (12-MTA), philinopside E, triterpene glycoside compounds, glycosaminoglycan, and chondroitin sulfates. These compounds are known to have antimicrobial, antioxidant, anti-inflammatory, and anti-tumor effects and can be used to treat lung cancer, pancreatic cancer, colorectal cancer, liver cancer, and more (Janakiram et al., 2015; Mayer et al., 2022).

#### 3.5.1 Echinoside A (EA)

Echinoside A (EA) is a triterpene glycoside, which is a major secondary metabolite of sea cucumbers (*P. graeffei* Semper). It has various pharmacological effects, such as enhancing immunity, being anti-cancer, anti-bacterial, and anti-angiogenesis (Zhao et al., 2018). Using 2.49 μmol/L of EA on HepG2 cells for 24 h can inhibit cell proliferation by more than 50%. Further research has found that EA can increase the expression level of tissue inhibitors of metalloproteinases (TIMP-1), downregulate the expression of MMP-9, reduce the MMP-9/TIMP-1 ratio, and protect the integrity of the extracellular matrix (ECM), thereby inhibiting tumor metastasis. At the same time, the downregulation of MMP-9 will also inhibit the expression of vascular endothelial growth factor (VEGF), inhibiting tumor angiogenesis (Zhao et al., 2011; Wang et al., 2014).

#### 3.5.2 Holothurin A1/24-dehydroechinoside A

Holothurin A1 (HA1) and 24-dehydroechinoside A (DHEA) are two sulfated triterpene glycosides isolated from sea cucumbers (*P. graeffei* Semper). Both 2.86 μM of HA1 and DHEA can reduce the average migration rate of HepG2 cells to 60% and 56.4%, respectively. Further research has found that both of them can directly inhibit the migration ability of HepG2 cells by reducing the expression of MMP-9 and increasing TIMP-1. At the same time, HA1 and DHEA can also downregulate the expression of VEGF in HCC cells, thereby inhibiting tumor cell growth (Zhao et al., 2010).

### 3.6 *Siphonochalina*

Sponges (*Siphonochalina*) are a class of primitive, multicellular aquatic animals. Since the 1960s, a series of compounds, such as spongothymidine, spongouridine, spongonucleoside, etc., have been isolated from sponges. They have high medicinal value, possessing antibacterial, anti-inflammatory, and antitumor effects (Ye et al., 2015; Elissawy et al., 2021).

#### 3.6.1 Sipholenol A

Sipholenol A is a triterpene derivative isolated from *Siphonochalina* sp. It has been proven to reverse P-gp-mediated MDR. It exhibits anti-proliferative activity in HepG2 cells. Sipholenol A can inhibit the expression of cyclin B1 and cyclin D1, upregulate the expression of Caspase-3, block cells in the G0/G1 and S phases, and induce cell apoptosis (Abdel-Lateff et al., 2016).

#### 3.6.2 Crambescidin-816

Crambescidin-816 (C816) is a guanidine alkaloid produced by the sponge *Crambe crambe,* with antitumor activity. It can reduce the viability of HepG2 cells by more than 50% and prevent cell migration. C816 can downregulate the expression of cyclin A and cyclin D as well as cyclin-dependent kinases 2 and 6, while increasing the expression of p53 and cyclin-dependent kinase inhibitors A and D, causing cell cycle arrest in the G0/G1 phase, and reducing the proportion of cells in the S and G2/M phases (Rubiolo et al., 2014).

#### 3.6.3 Aaptamine

Aaptamine is an alkaloid extracted from the sponge *Aaptos aaptos* Schmidt. It is a potential antimicrobial drug and also has good antitumor effects (Tsukamoto et al., 2010; Nadar et al., 2022). Aaptamine can inhibit the growth and colony formation of HCC-LM3 and HepG2 cells. Further research found that aaptamine reduces the expression of SRY-box transcription factor 9 and CDK2 in HCC cells, inhibits CDK2 kinase activity, and promotes the expression of p21 and the binding of the CDK2-cyclin D/E complex, thereby inhibiting the cell cycle. *In vivo* experiments also confirmed the antitumor effect of aaptamine in tumor-bearing mice (Li et al., 2015).

### 3.7 Alcyoniidae

Soft corals (Alcyoniidae) are a class of lower invertebrates. Extracts from corals have anti-inflammatory, anti-tumor, tissue repair, neuroprotective, and antihypertensive effects. A variety of compounds, such as diterpenoids, diterpenes, and prostanoids, have been isolated from corals, which have certain antitumor effects on liver cancer, breast cancer, colon cancer, and cervical cancer (Kamel et al., 2007; Poza et al., 2008). Among them, crassarosterol A, crassarosterol C, juncenolides C, Australins B, etc., all show anti-HCC activity (Shen et al., 2003; Ahmed et al., 2005; Chao et al., 2012).

#### 3.7.1 Bromovulone III

Bromovulone III is a marine prostanoid compound isolated from the soft coral *Clavularia viridis* Quoy & Gaimard. The IC50s for Hep3B, HepG2, and HA22T cells are 0.47 ± 0.02, 0.59, and 0.41 mM, respectively. Mechanistic studies have shown that bromovulone III induces apoptosis in HepG3B cells by inducing m-calpain and Caspase-7/12 activation, causing endoplasmic reticulum (ER) stress and increased expression of CHOP/GADD153, leading to secondary mitochondrial swelling and mitochondrial membrane depolarization (Chiang et al., 2005).

#### 3.7.2 Methyl spongoate

Methyl spongoate (MESP) is a methyl spongoate analog that has a certain killing effect on BEL-7402, SMMC-7221, Zip-177, HepG3B, HepG2, and BEL-7404 cells, with an average IC50 of 4.4 µM (Jiang et al., 2010). Mechanistic studies have shown that MESP induces typical apoptosis by disrupting MMPs and activating the Caspase-9/3 cascade. On the other hand, it induces cell apoptosis by alleviating the inhibition of the STAT3 signaling pathway, reducing the expression of the anti-apoptotic x-linked inhibitor of apoptosis protein, and promoting the expression of BAX and Caspase-3/9 (Jiang et al., 2011).

#### 3.7.3 11-epi-sinulariolide acetate

11-epi-sinulariolide acetate (11-epi-SA) is an active compound isolated from *Sinularia flexibilis* Quoy & Gaimard, which also has anti-HCC effects. 15.9 μM of 11-epi-SA can significantly inhibit the proliferation and migration of HA22T cells, inhibit the protein levels of MMP-2, MMP-9, and urokinase-type plasminogen activator (uPA) in cells, increase the expression of TIMP-1 and TIMP-2, and inhibit the phosphorylation of extracellular signal-regulated kinase 1/2 (ERK1/2) and p38 MAPK and the focal adhesion kinase (FAK)/PI3K/Akt/mTOR pathway, thereby inhibiting HCC (Lin et al., 2014). In addition, 11-epi-SA can induce apoptosis through the mitochondrial pathway by upregulating BAX and Bcl2-associated agonist of cell death (BAD), downregulating BCL-2, and inducing apoptosis by causing endoplasmic reticulum stress and upregulating CHOP expression (Lin et al., 2016).

### 3.8 Other animal-derived natural products

#### 3.8.1 Extractive of *Periplaneta american* Linneaus

The American cockroach belongs to the class Insecta, family Blattidae, and genus *Periplaneta*. It has been used in traditional Chinese medicine for thousands of years. Modern research shows that it has anti-tumor and anti-inflammatory effects. In China, a variety of drugs have been developed using the American cockroach as a raw material, which has significant effects on the treatment of liver diseases (Ganlong capsule and Xiaozheng Yigan tablet). Research has found that the extract of Periplaneta american L. at 0.15624 mg/mL can exert anti-HCC effects by regulating the P13K/Akt pathway (Ma et al., 2022).

#### 3.8.2 Kahalalide F

Kahalalide F (KF) is an active ingredient extracted from the marine mollusk *Elysia rufescens*, showing a variety of antitumor effects. After KF treatment, the growth of HepG2, Huh-7, and Hep2B cells is inhibited, and the cell cycle is arrested in the G0-G1 phase, which is related to the inhibition of TGF-α expression and the blockade of the epidermal growth factor receptor. Clinical trials have also shown the beneficial effects of KF. After receiving KF treatment, the condition of HCC patients can remain stable for a long time (Pardo et al., 2008; Salazar et al., 2013).

#### 3.8.3 β-chitosan nanoparticles

β-chitosan nanoparticles (β-CNP) is a chitosan-like component extracted from the cuttlebone of cephalopods, which has a certain antibacterial effect. β-CNP synthesized using sodium tripolyphosphate and β-chitosan as raw materials show good anticancer activity. 30 μg/mL of β-CNP can promote apoptosis of HepG2 cells in a concentration-dependent manner through the mitochondrial pathway (Subhapradha and Shanmugam, 2017).

**FIGURE 2:**
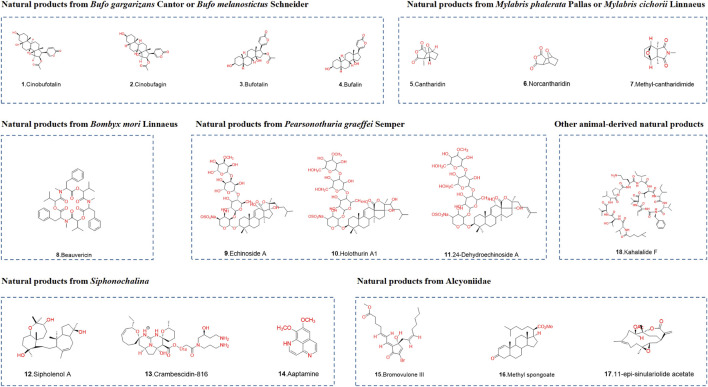
Chemical structures of animal-derived natural products. Classification of animal-derived natural products according to species.

The structures of animal-derived natural products are shown in Figure 2.

## 4 Discussion and perspective

### 4.1 Mechanism of action

#### 4.1.1 Wnt/β-catenin pathway

Under physiological conditions, the wnt pathway is inhibited and the GSK complex phosphorylates β-catenin, leading to its degradation in the cytoplasm. The over-activation of the Wnt pathway in cancer results in Wnt binding to low-density lipoprotein receptor-related proteins 5/6 and other molecules. This causes the GSK complex to lose its inhibition of β-catenin, allowing β-catenin to enter the nucleus and interact with the transcription factors to activate downstream transcription. The primary downstream molecules of β-catenin are N-cadherin, vimentin, slug, and snail (Zhang X. et al., 2020). Over-transcription of these molecules prevents E-cadherin from mediating intercellular tight junctions and encourages EMT, the primary mechanism through which cancer cells undergo migratory invasion.

#### 4.1.2 Caspase apoptosis pathway

Apoptosis is typified by DNA fragmentation, nuclear fixation, cell shrinkage, and blebbing of the cell membrane. There are two types of pathways: internal (mitochondrial) and external. The apoptosis receptor Fas, TRAIL-R, binds to its ligand Fas-L, TRAIL, causing Fas-associated death domain protein (FADD) to undergo a conformational change that changes procaspase8/10 into Caspase8/10. Then, the downstream Caspase3/7 is activated by the external pathway, and apoptosis is induced by the internal pathway via the mitochondrial pathway. Activation of the mitochondrial pathway can also be achieved by activating BAX and Bcl2 antagonist/killer 1 (BAK) to inhibit the BCL-2 apoptosis suppressor family proteins. Increased mitochondrial permeability releases cytochrome C into the cytoplasm, where it binds to proteins like procaspase-9 to form apoptotic vesicles. Procaspase-9 is then cleaved to form enzymatically active Caspase-9, which starts the Caspase cascade reaction and eventually activates PARP cleavage and apoptosis.

#### 4.1.3 p53/BCL-2/BAX pathway

p53 can control cell division and stop the development of HCC tumor cells by stopping cells with damaged or mutant DNA from proliferating and by transcriptionally sending these cells death signals (Ji et al., 2022). p53 can activate p21, growth arrest, and the DNA damage-inducible protein GADD45 alpha, etc. to stop the HCC cell cycle; it can also upregulate apoptotic protease-activating factor 1, BAX, Fas, etc. to encourage apoptosis in HCC cells; it can also promote angiogenesis by upregulating thrombospondin-1, etc.; it can also increase the activity of 5′-AMP-activated protein kinase subunit beta-1, Bcl2 binding component 3 (PUMA), etc. to encourage autophagy; as well as can activate DNA damage-binding protein 2, fanconi anemia group C protein, etc. to promote DNA repair.

The E3 ubiquitin-protein ligase mdm2 (MDM2) and the protein mdm4 (MDMX), which is a component of p53, prevent P53 activation, whereas the tumour suppressor ARF inhibits MDM2 (Cinelli et al., 1998). The transcription of the oncogene’s downstream target genes is induced when tumor suppressor ARF activity is triggered, MDM2 is repressed, and p53 is activated. p53 is involved in a number of processes, including angiogenesis, apoptosis, cell cycle disruption, autophagy stimulation, and DNA repair.

#### 4.1.4 PI3K/Akt pathway

The overactivation of the PI3K/Akt pathway typically results in malignant invasion and medication resistance in tumor cells (Khezri et al., 2022). PI3K is activated by adaptor proteins, growth factors, and other molecules that catalyze the formation of PIP3 from PIP2, activating Akt. phosphatidylinositol 3,4,5-trisphosphate 3-phosphatase and dual-specificity protein phosphatase PTEN (PTEN) converts PIP3 to PIP2 and inhibits PI3K/Akt. Akt activation stimulates multiple downstream pathways. For instance, PI3K triggers mTOR, which subsequently prevents ATG1-mediated autophagy. Moreover, BAD is phosphorylated by Akt, stimulating BCL2 and preventing apoptosis. By phosphorylating MDM2, Akt blocks the p53 pathway. Additionally, p21 and p27 are phosphorylated by Akt, which inhibits CDK and stops the cell cycle. When GSK is inactivated by Akt, β-catenin translocates to the nucleus and triggers downstream transcription. Additionally, Akt blocks the MARK pathway (Song et al., 2019).

#### 4.1.5 NF-κB pathway

Ageing, chronic inflammatory disorders, tumorigenesis, and autoimmune diseases are all significantly impacted by the NF-κB signaling system (Hoesel and Schmid, 2013). New research indicates that liver fibrosis and HCC may be prevented or treated by targeting it (Luedde and Schwabe, 2011). NF-κB is made up of nuclear factor p50 and p65. IκB kinase (IKK) phosphorylates and degrades IκB, allowing NF-κB to enter the nucleus and activate the downstream genes. Through the activation of IKK, Akt can initiate the NF-κB pathway. In tumor-associated macrophages, dendritic cells, myeloid-derived suppressor cells, and natural killer cell cells, NF-κB activation may be involved in controlling inflammation, carcinogenesis, and migration. To improve autophagy, for instance, NF-κB activates beclin-1 and other autophagy-related proteins.

#### 4.1.6 HIF-1α and VEGF pathway

Hypoxia-inducible factor 1-alpha (HIF-1**α**) is composed of an O2-regulated HIF-1α subunit and a constitutively-expressed HIF-1β subunit. Prolyl hydroxylase is triggered in an aerobic environment, which leads to the degradation of HIF-1α (Gao et al., 2023). Hypoxia causes the α and β subunits to translocate to the nucleus, where they bind to the cofactor P300. This triggers downstream mechanisms that include boosting the release of cytokines, controlling ECM remodeling and metabolism to support cell survival, regulating VEGF, and inducing angiogenesis. In the meantime, transduction of the mTOR, STAT3, and NF-κB signaling pathways can increase its expression (Chen et al., 2019).

In cancer, the vascular endothelial growth factor receptor (VEGFR) family of receptors have three varieties: 1, 2, and 3. VEGFR2 is the most prevalent variety. After binding to VEGF, VEGFR2 undergoes autophosphorylation, triggering the signaling pathways of ERK/MAPK, calcium, and PI3K/Akt to become active. The PI3K/Akt signaling pathway increases the survival of vascular endothelial cells by inhibiting BAD and Caspase 9. In the meantime, nitric oxide synthase 3 is activated, and nitric oxide levels are raised via the PI3K/Akt and calcium ion signaling pathways, which increase vascular permeability. Furthermore, the MAPK and PI3K/Akt pathways facilitate cell invasion and migration. The ERK/MAPK pathway then triggers cell division.

**FIGURE 3:**
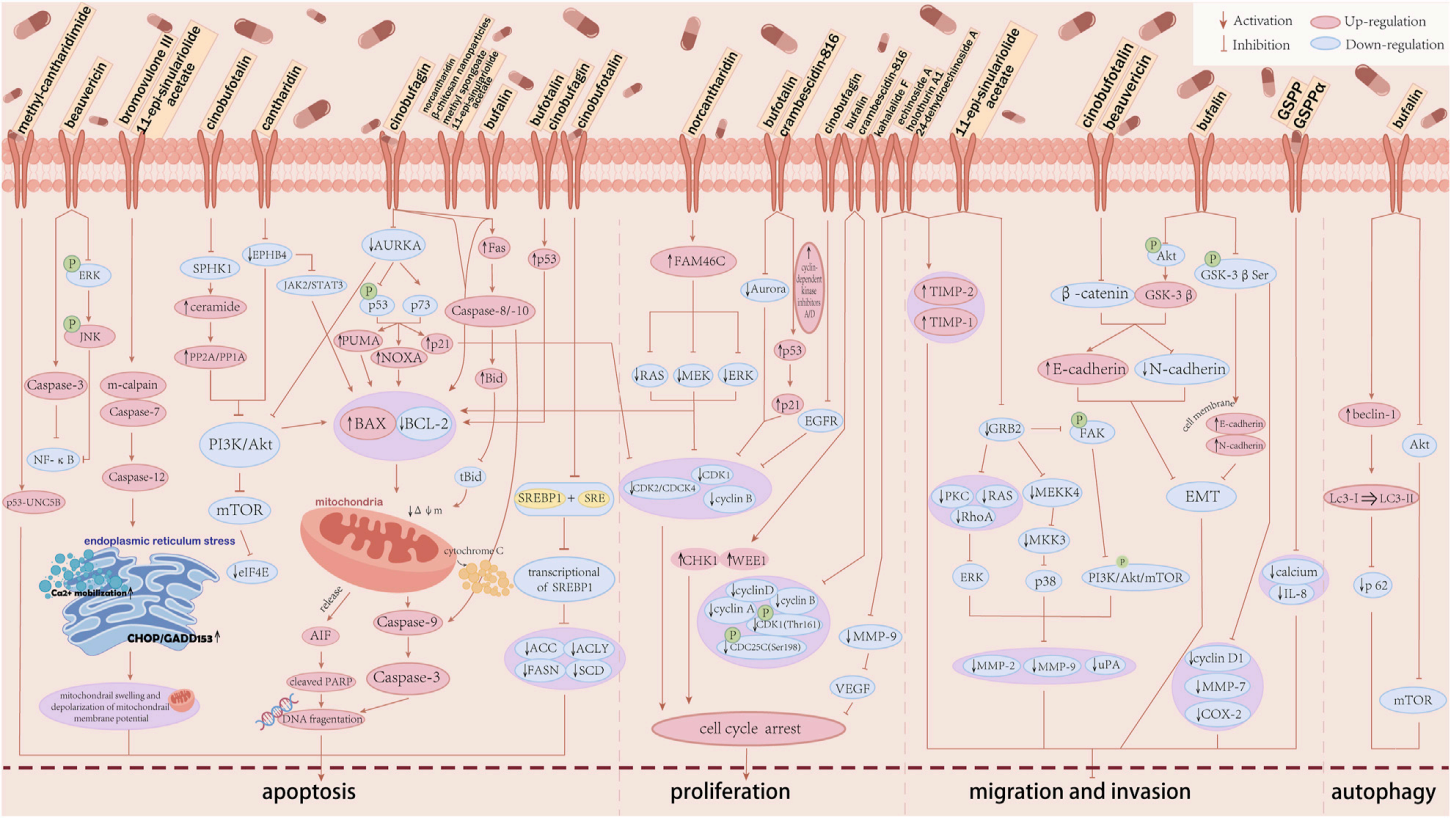
The anti-hepatocellular carcinoma mechanism of animal-derived natural products. The anti-hepatocellular carcinoma activity of each animal-derived natural product is mainly achieved by inducing apoptosis in HCC, blocking the cell cycle, inhibiting invasion and migration, and inducing autophagy. ACC, acetyl-CoA carboxylase; ACLY, ATP citrate lyase; Akt, protein kinase B; AURKA, aurora kinase A; BAX, Bcl2 associated X; BCL-2, Bcl2 apoptosis regulator; CDC, cell division cycle; CDK, cyclin-dependent kinase; CHK1, checkpoint kinase 1; EGFR, epidermal growth factor receptor; eIF4E, eukaryotic translation initiation factor 4E; EMT, epithelial-mesenchymal transition; EPHB4, ephrin type-B receptor 4; ERK, extracellular signal regulated kinases; ERK1/2, extracellular signal-regulated kinase 1/2; FAM46C, terminal nucleotidyltransferase 5C; FAK, focal adhesion kinase; FASN, fatty acid synthase; GRB2, growth factor receptor-bound protein 2; GSK3β, glycogen synthase kinase-3 beta; IL-8, interleukin-8; JAK2, Janus kinase2; JNK, c-Jun N-terminal kinase; Lc3, light chain-3; MAPK, mitogen-activated protein kinase; MDR, multidrug resistance; MEK, mitogen-activated protein kinase 1; MEKK4, MAPK kinase kinasekinase4; MKK3, mitogen-activated protein kinase3; MMPs, matrix metalloproteinase; mTOR, mammalian target of rapamycin; NOXA, phorbol-12-myristate-13-acetate-induced protein 1; NF-κB, nuclear factor-κB; p, phosphorylation; PARP, poly ADP-ribose polymerase; PI3K, phosphoinositide 3-kinase; PKC, anti-humanprotein kinase C; PP, serine/threonine phosphatases; PUMA, Bcl2 binding component 3; p21, cyclin-dependent kinase inhibitor 1; SCD1, Stearoyl-CoA desaturase-1; SPHK1, sphingosine kinase 1; SRE, sterol regulatory elements; SREBF1, sterol regulatory element-binding protein 1; STAT3, signal transducer and activator of transcription 3; TIMP-1/2, tissue inhibitor of metalloproteinase-1/2; UNC5B, netrin receptor UNC5B; uPA, urokinase-type plasminogen activator; VEGF, vascular endothelial growth factor; WEE1, wee1-like protein kinase; ΔΦm, mitochondrial membrane potential.

The specific anti-HCC mechanistic pathways of animal-derived natural products are summarised in Figure 3.

### 4.2 Drug toxicity

Animal medicines have shown great potential in cancer treatment, but their toxicity greatly limits their application. The main toxicities are blood toxicity, alopecia, neurotoxicity, and cardiac toxicity. the lethal dose of CTD for human oral administration is 10–60 mg (Wang and Yang, 2019). Many case reports have recorded injuries caused by the misuse of animal medicines. A 51-year-old male showed symptoms such as oral burning, diarrhea, difficulty urinating, hematuria, and acute kidney injury after taking an aphrodisiac containing CTD (Diaz et al., 2020). Four males were poisoned after taking CTD, showing gastrointestinal reactions and liver and kidney function damage (Karras et al., 1996). It is reported that from 1996 to 2016, there were 151 cases of animal medicine poisoning in China, with a mortality rate of 18.68% (Helman and Edwards, 1997). *In vivo* studies, we discovered that injections of huachansu resulted in aberrant alterations in myocardial damage indices (AST, CK-MB, and cTnI) following administration and ventricular tachycardia in Beagle dogs. Myocardial damage indices (cTnI) in SD rats similarly displayed aberrant alterations following dosing (Min, 2020). Mice injected with KF displayed both potentially lethal neurotoxicity and reversible nephrotoxicity. Through clinical trials, we discovered that CTD usage had severe harmful side effects on various systems in addition to its high hepatotoxicity. Toxic side effects are evident and include bleeding into the stomach, myocardial ischaemia, respiratory distress, renal failure, and more (Zhang and Zhang, 2020).

It is necessary to inhibit its toxicity while exerting the anticancer effect of animal medicine. In China, researchers have developed a variety of animal extract preparations, purified the medicinal components, and removed toxic components, such as Huachansu injection, Jinlong capsule, etc. In addition, it is promising to develop new drugs with anticancer effects and low toxicity characteristics by modifying certain structures according to the chemical structure and structure-effect characteristics of animal active ingredients. A study prepared 4 derivatives based on the structural characteristics of toad dienolide, confirming the impact of structural modification on drug cytotoxicity (Cunha-Filho et al., 2010). Recently, machine learning seems to greatly accelerate the process of optimizing drug structure (Janela and Bajorath, 2022), which is worth the attention of pharmacists. On the other hand, nanoformulations can also optimize some properties of animal medicines, such as toxicity, oral utilization, organ targeting, etc. Researchers used long-cycle nanoparticle liposomes to package and deliver bufalin, which improved the retention of the drug in the body, and this nanoformulation was less distributed in the heart and kidney organs, which was more conducive to drug enrichment and beneficial to the treatment of liver and lung diseases (Ai-ping, 2020). Mesenchymal stem cell-derived exosomes (MSC-Exos) have intrinsic therapeutic characteristics to reduce liver tissue damage. Researchers use this characteristic to use bone mesenchymal stem cell-derived exosomes (BMSC-Exos) as drug carriers to encase the anticancer drug NCTD. *In vitro* experiments found that BMSC-Exos-NCTD can slow down drug release, effectively promote cell uptake, reduce tumor cell proliferation, induce cell cycle arrest, and increase cell apoptosis compared to simple NCTD treatment. *In vivo* experiments found that BMSC-Exos carriers have an *in situ* homing effect on the tumor site of mouse HCC and did not show toxicity (Liang et al., 2021).

### 4.3 Combination therapy

At present, various chemotherapies and small molecule therapies are still the most important methods for treating HCC. Animal active ingredients may be combined with these main treatment methods to achieve unexpected good effects. Crizotinib has been approved by the FDA for use in malignant tumors with anaplastic lymphoma kinase (ALK) mutations. The combined use of NCTD and crizotinib can significantly inhibit the growth of tumors in tumor-bearing mice, and the effect is better than that of a single drug, which may be related to NCTD inhibiting the mTOR pathway (Sun et al., 2017). Sorafenib is a common drug for the treatment of advanced HCC. A clinical study in China used sorafenib and toad extract (Huamao) to treat HCC and obtained a higher effective rate than a single drug, and the combined group had more significant improvements in WBC, TBIL, and ALT indicators, a higher quality of life for patients, and weaker pain (Feng et al., 2012). The combined use of sorafenib and sodium cantharidinate can enhance the inhibition of HepG2 cell proliferation, significantly block cell proliferation in the G1 phase, and its mechanism may be related to the inhibition of the Ras/MEK/ERK pathway (CHEN et al., 2015).

Some natural products derived from plants seem to also enhance the effects of animal-derived products. Kanglaite injection is an anti-tumor drug extracted from the Semen Coicis (*Coix lacryma-jobi* var. *ma-yuen* (Rom. Caill.) Stapf), which can effectively inhibit the cell cycle of tumor cells. The combination of kanglaite injection and cinobufacin tablets has a definite therapeutic effect on patients with HCC. Compared with single-drug use, combined medication can relieve patient pain better, and have no obvious adverse reactions (ke, 2018). Evodiamine (EVO) is one of the extracts from *Evodia rutaecarpa* (Juss.) Benth., which has anti-cancer and anti-inflammatory effects. *In vitro* experiments found that EVO combined with NCTD showed a synergistic effect of anti-proliferation and apoptosis in liver cancer cells (Liu and You, 2014). Its mechanism may be to inhibit the growth of HepG2 cells by reducing the BAX/BCL-2 ratio, blocking the cell cycle in the G2/M phase, and increasing the level of reactive oxygen species in cells (yumin, 2015). Triptolide is a diterpene epoxide extracted from *Tripterygium wilfordii* Hook. F. The combined use of sodium cantharidinate and triptolide can synergistically inhibit the growth of 7,721 cells. Its mechanism is related to increasing Caspase-3 activity and suppressing NF-κB (Zhou and Pan, 2016).

The results of using contemporary medical technologies in combination with animal medication therapy are likewise favorable. Ultrasound-guided percutaneous hepatic portal vein puncture injection of Huachansu was used in 25 patients with HCC and portal vein thrombosis. The treatment’s overall effectiveness rate was 68% (Jianfeng et al., 2015). 16 cases of HCC were treated with transcatheter arterial chemoembolization (TACE) in conjunction with percutaneous portal vein cannulation for a continuous infusion of Huachansu injection. Following treatment, the tumors were completely necrotic in 6 cases (37.5%), significantly shrunk in 5 cases (31.25%), and stable in 2 cases (12.5%) (Chen and Wu, 2010). The combination of drugs in the experimental group increased the rate of tumor remission, improved quality of life, and increased 1-year survival in a meta-analysis of huachansu injection plus hepatic artery chemoembolization for primary hepatocellular carcinoma (Ding et al., 2020). CTD can also be used in the post-operative phase to enhance patients’ immunity. Eighty patients with primary liver cancer were randomly assigned to one of two groups in a clinical trial: the test group (TACE + CTD compound capsule treatment) or the control group (TACE treatment). The results indicated that the test group had a higher rate of solid tumor control than the control group (Zhao S. C. et al., 2019).

### 4.4 Animal protection

Undoubtedly, the production of animal medicines depends on live animals, which forces us to consider animal ethics and animal protection when applying animal medicines on a large scale. In the past, many animals have been hunted on a large scale due to their unique medicinal properties, leading to endangered or extinct animals. Therefore, it is necessary to study the ecological characteristics of the source animals before researching a certain animal-derived products to avoid looking for new drugs in animals that are difficult to artificially breed or have a small population. In addition, humane treatment of animals during the drug acquisition process is also necessary.

With the development of pharmaceutical chemistry, it is feasible to artificially synthesize animal-derived drugs with known structures. In China, some rare animal-derived traditional medicines, such as bezoar and musk, have been mass-produced through artificial synthesis. These drugs have effects that are not inferior to real animal extracts, and they avoid the ecological and human crises caused by slaughtering animals.

## 5 Conclusion

HCC is a malignant tumor that seriously threatens human life and health, with its incidence and mortality rates remaining high worldwide. Due to the difficulty of early detection and late-stage treatment, the cure rate for HCC is extremely low. Active ingredients derived from animals have a long history, and many animal medicines have been used to treat HCC in traditional medicine. In recent years, research on active ingredients from animals has gradually increased, providing more possibilities for the discovery of new anti-HCC drugs.

This review summarizes the animal-derived natural products that have been found to have potential anti-HCC effects, such as cinobufotalin, cinobufagin, bufalin, CTD, NCTD, MCA, beauvericin, etc. These animal-derived products induce cell apoptosis, induce autophagy, reverse drug resistance, inhibit tumor migration, block the cell cycle, and inhibit tumor cell metabolism through various pathways such as PI3K/Akt/mTOR, Ras/ERK/MAPK, Wnt/β-catenin, JAK/STAT, etc., and have extremely high clinical potential.

However, there are still many obstacles to the clinical application of animal-derived products. The components and mechanisms of many animal-derived products are still unclear and need further research. At the same time, the toxicity and side effects of animal-derived products need to be given special attention, and drugs with better effects and lower toxicity need to be developed through methods such as optimizing drug structure and preparing nanoformulations. Animal ethics and animal protection are also issues that need to be considered before applying animal-derived products. Despite these obstacles, there is no doubt that animals, like plants, are important avenues for the discovery of new drugs and are worthy of researchers’ attention.

## Figures and Tables

**Table:** 

**ACC**	acetyl-CoA carboxylase
**ACLY**	ATP citrate lyase
**AFP**	alpha-fetoprotein
**ALB**	Albumin
**ALK**	anaplastic lymphoma kinase
**Akt**	protein kinase B
**AURKA**	aurora kinase A
**BAD**	Bcl2-associated agonist of cell death
**BAK**	Bcl2 antagonist/killer 1
**BAX**	Bcl2 associated X
**BCL-2**	Bcl2 apoptosis regulator
**BMSC-Exos**	bone mesenchymal stem cell-derived exosomes
**Caspase**	cysteine-dependent aspartate-directed proteases
**CDC**	cell division cycle
**CDK**	cyclin-dependent kinase
**CHK1**	checkpoint kinase 1
**CTD**	cantharidin
**c-Met**	the mesenchymal-epithelial transition factor
**C816**	crambescidin-816
**DHEA**	24-Dehydroechinoside A
**EA**	Echinoside A
**EGFR**	epidermal growth factor receptor
**ECM**	extracellular matrix
**EIF4E**	eukaryotic translation initiation factor 4E
**EMT**	epithelial-mesenchymal transition
**EPHB4**	ephrin type-B receptor 4
**ER**	endoplasmic reticulum
**ERK**	extracellular signal regulated kinases
**ERK1/2**	extracellular signal-regulated kinase 1/2
**EVO**	Evodiamine
**FADD**	Fas-associated death domain protein
**FAM46C**	terminal nucleotidyltransferase 5C
**FAK**	focal adhesion kinase
**FASN**	fatty acid synthase
**Gepsin**	Gekko sulfated polysaccha-ride
**GRB2**	growth factor receptor-bound protein 2
**GSPP**	Gekko swinhonissulfated polysaccharide-protein complex
**GSPPα**	Gekko sulfated glycopeptide α
**GSK3**	glycogen synthase kinase-3
**HA1**	Holothurin A1
**HCC**	Hepatocellular carcinoma
**HIF-1α**	hypoxia-inducible factor 1-alpha
**IKK**	IκB kinase
**IL-8**	interleukin-8
**JAK**	Janus kinase
**JAK2**	Janus kinase2
**JNK**	c-Jun N-terminal kinase
**KF**	Kahalalide F
**Lc3**	light chain-3
**MAPK**	mitogen-activated protein kinase
**MCA**	methyl-cantharidimide
**MDR**	multidrug resistance
**MDM2**	E3 ubiquitin-protein ligase Mdm2
**MDMX**	protein Mdm4
**MEK**	mitogen-activated protein kinase
**MEKK4**	MAPK kinase kinasekinase4
**MESP**	Methyl spongoate
**MKK3**	mitogen-activated protein kinase3
**MMPs**	matrix metalloproteinase
**MSC-Exos**	Mesenchymal stem cell-derived exosomes
**mTOR**	mammalian target of rapamycin
**NCTD**	norcantharidin
**NOXA**	phorbol-12-myristate-13-acetate-induced protein 1
**NF-κB**	nuclear factor-κB
**PARP**	poly ADP-ribose polymerase
**p-gy**	p-glycoprotein
**PI3K**	phosphoinositide 3-kinase
**PKC**	anti-humanprotein kinase C
**PP**	serine/threonine phosphatases
**PUMA**	Bcl2 binding component 3
**p21**	cyclin-dependent kinase inhibitor 1
**p62**	sequestosome-1
**SCD1**	Stearoyl-CoA desaturase-1
**SPHK1**	sphingosine kinase 1
**SRE**	sterol regulatory elements
**SREBF1**	sterol regulatory element-binding protein 1
**STAT**	signal transducer and activator of transcription
**STAT3**	signal transducer and activator of transcription 3
**TACE**	transcatheter arterial chemoembolization
**TIMP-1/2**	tissue inhibitor of metalloproteinase-1/2
**TNF**	tumor necrosis factor
**TRAIL**	TNF-related apoptosis-inducing ligand
**UNC5B**	netrin receptor UNC5B
**UPA**	urokinase-type plasminogen activator
**VEGF**	vascular endothelial growth factor
**VEGFR**	vascular endothelial growth factor receptor
**WEE1**	wee1-like protein kinase
**11-epi-SA**	11-epi-sinulariolide acetate
**12-MTA**	12-methyltetradecanoic acid
**β-CNP**	β-chitosan nanoparticles
**ΔΦm**	mitochondrial membrane potential
